# Supplementary material for: Assessing Protein Surface-Based Scoring for Interpreting Genomic Variants
Source: Int J Mol Sci. 2024 Nov 8;25(22):12018. doi: 10.3390/ijms252212018 (PMC11594063; doi:10.3390/ijms252212018)
Supplement: Supplementary file 1 [file ijms-25-12018-s001.zip › ijms-3251455-supplementary.pdf]

Supplementary material for "Assessing Protein Surface-Based Scoring for Interpreting Genomic Variants"

Authors: Nikita R. Dsouza[1], Neshatul Haque[1], Swarnendu Tripathi[1,2], Michael T. Zimmermann[1,3,4,*]

[1]Computational Structural Genomics Unit, Linda T. and John A. Mellowes Center for Genomics Sciences and Precision Medicine, Medical College of Wisconsin, Milwaukee, WI 53226, USA

[2]Current address: St. Jude Children's Research Hospital, Memphis, TN 38105, USA

[3]Clinical and Translational Sciences Institute, Medical College of Wisconsin, Milwaukee, WI 53226, USA

[4]Department of Biochemistry, Medical College of Wisconsin, Milwaukee, WI 53226, USA

[*]Corresponding Author

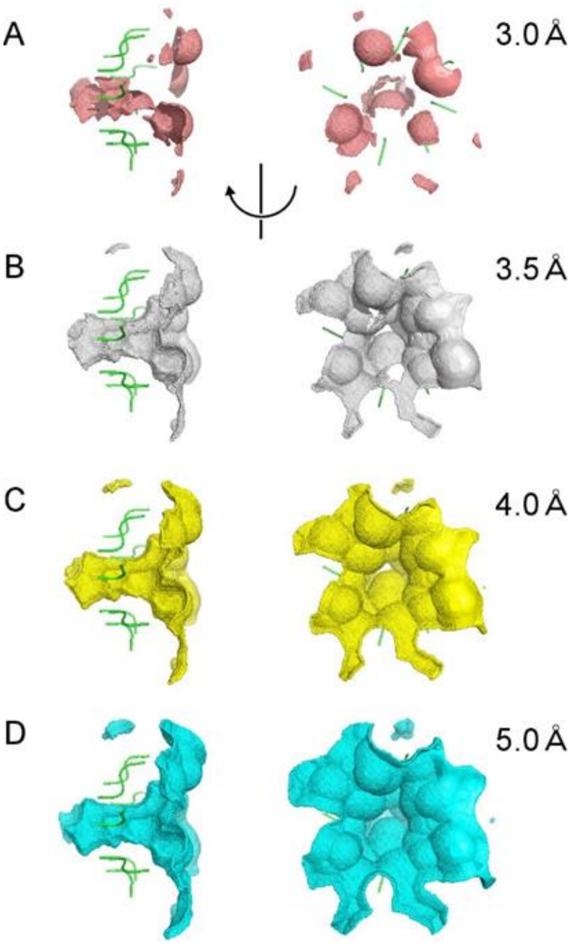

Supplemental Data

Supplemental Figure S1: Selection of the surface subset and distance. Using TBL1XR1's binding interface, we tested different length cutoffs for how well they describe a specific protein surface region. A) At 3.0Å cutoff, too little of the surface in captures. B) Similarly at 3.5Å. C) By 4Å, the whole interaction surface is captures, but at D) 5Å, we begin to capture more of the surrounding non-interface. Thus, in this study, we used 4Å as a cutoff to select the local surface, including the variant and the surrounding protein environment.
